# Assessing the Efficacy of a 28-Day Comprehensive Online Prostate Cancer Patient Empowerment Program (PC-PEP) in Facilitating Engagement of Prostate Cancer Patients in Their Survivorship Care: A Qualitative Study

**DOI:** 10.3390/curroncol30090626

**Published:** 2023-09-21

**Authors:** Gabriela Ilie, Cody MacDonald, Hal Richman, Ricardo Rendon, Ross Mason, Alexandra Nuyens, Greg Bailly, David Bell, Nikhilesh Patil, David Bowes, Emmi Champion, Derek Wilke, Lia Massoeurs, Nada Hassan, Robert David Harold Rutledge

**Affiliations:** 1 Department of Community Health and Epidemiology, Faculty of Medicine, Dalhousie University, Halifax, NS B3H 4R2, Canada; codymacdonald@dal.ca (C.M.); hrichman12@gmail.com (H.R.); alexandra.nuyens@dal.ca (A.N.); lia.massoeurs@dal.ca (L.M.); nd424678@dal.ca (N.H.); 2 Department of Urology, Faculty of Medicine, Dalhousie University, Halifax, NS B3H 4R2, Canada; 3 Department of Radiation Oncology, Faculty of Medicine, Dalhousie University, Halifax, NS B3H 4R2, Canada; 4 IWK Health Centre, Halifax, NS B3K 6R8, Canada

**Keywords:** prostate cancer, survivorship, patient education, patient advocacy, patient empowerment, patient engagement, patient activation, cancer survivorship, quality of life, mental health

## Abstract

A 28-day Prostate Cancer-Patient Empowerment Program (PC-PEP) developed through patient engagement was successful at promoting mental and physical health. Thirty prostate cancer patients from Halifax, Canada participated in the 28-day PC-PEP intervention in early 2019. PC-PEP encompassed daily patient education and empowerment videos, prescribed physical activities (including pelvic floor exercises), a mostly plant-based diet, stress reduction techniques, intimacy education, social connection, and support. Quantitative exit surveys and semi-structured interviews (conducted in focus groups of ten) were used to assess perceived factors that facilitated or impeded adherence to the program. The program received high praise from the patients and was deemed extremely useful by the participating men, who rated it 9 out of 10. Patients expressed that the multifaceted, online, home-based nature of the program helped them adhere to it better than they would have had to a single or less comprehensive intervention. Feedback from the participants indicated that the program, when viewed as a whole, was perceived as greater than the sum of its individual parts. Furthermore, the program addressed various issues, including emotional vulnerability and distress, physical fitness, urinary incontinence, challenges in expressing emotions, perceived lack of control over healthcare decisions, emotional fragility, and hesitancy to discuss prostate cancer-related matters in social settings. Patients highly (9.6/10) endorsed integrating the program into the standard care regimen from the very beginning of diagnosis. However, challenges such as work commitments were noted. Patients’ high endorsement of PC-PEP suggests that its implementation into the standard of care from day one of diagnosis may be warranted.

## 1. Introduction

Prostate cancer, the most frequently diagnosed cancer among North American men, has witnessed substantial advancements in both detection and treatment over the past few decades [1,2]. Consequently, the survival rates for individuals diagnosed with localized prostate cancer have reached as high as 90% after a span of ten years from diagnosis [3,4]. Patients who have experienced the spread of cancer to other sites either before or after treatment can also anticipate long-term survivorship [5]. Acute and long-term side effects from active forms of treatment, however, remain a common concern among patients. Men with prostate cancer, often experience urinary issues, pain, and sexual dysfunction, which tend to co-occur with depression, psychological distress, anxiety, and negatively impact their quality of life [6,7,8,9]. Yet, interventions aimed at reducing psychosocial and physical side effects from treatments and treatments-related side effects among prostate cancer patients and survivors remain sparse [10,11].

Expert panels focused on cancer survivorship have underscored the urgent need for evidence-based interventions that empower and educate prostate cancer survivors to address their psychosocial and mental health needs [12]. Patient education plays a vital role in preparing individuals to manage treatment-related side effects through lifestyle changes, skill development, and coping mechanisms [12,13,14,15,16]. To this end, prominent organizations such as the Canadian and American Cancer Societies, Urological Associations, and clinical and patient oncological support groups allocate significant resources to create reliable content for cancer patients and survivors [12,13]. In line with these efforts, the American Society of Clinical Oncology recognizes that informed patients tend to be more engaged in their care and experience fewer treatment-related quality-of-life issues [13,17].

Research aimed at uncovering the unmet needs of both prostate cancer patients and survivors has identified several substantial areas in need of support. These encompass critical aspects such as mental well-being, psychosocial factors, physical health, patient empowerment, and comprehensive health system information [18,19,20,21]. Studies have reported that between 33% and 81% of prostate cancer survivors describe having unmet needs and high mental distress during their survivorship journey [15,16,17,18,19,20,21,22].

Psychological distress among prostate cancer patients affects one in six patients and is often underdiagnosed and untreated [19,20,23,24]. The National Comprehensive Cancer Network (NCCN) defines cancer circumstantial psychological distress as: “a multifactorial unpleasant emotional experience of a psychological (cognitive, behavioral, emotional), social and/or spiritual nature that may interfere with the ability to cope effectively with cancer, its physical symptoms and its treatment…” [25,26]. When psychological distress remains unrecognized and is not clinically treated, it can have long-lasting effects and may lead to depression, isolation, existential crisis, poor quality of life, treatment regret, and poor patient recovery [27,28]. Conversely, effectively addressing the support needs of cancer patients during their journey can lead to improved mental health, enhanced quality of life, better communication, and reduced healthcare utilization [29,30,31]. Therefore, identifying and managing psychological distress in prostate cancer patients is of paramount importance [19,20,23,24,29,31,32].

Although education and empowerment interventions to support men diagnosed with prostate cancer in addressing these needs are emerging, they remain relatively scarce [11,29,30,31]. Recent years have witnessed significant efforts to systematically review the field of prostate cancer survivorship, particularly focusing on psychosocial and psychosexual interventions [11,33,34]. For instance, a 2017 study highlighted the effectiveness of multimodal interventions for men with localized prostate cancer, encompassing elements such as education, cognitive-behavioral therapy, communication, and peer support [33]. However, it also emphasized limitations in existing research, such as small sample sizes and methodological issues, underlining the necessity for further investigation into long-term survivorship outcomes. This study stressed the importance of considering sociodemographic and psychosocial variables when designing care and recognizing the need for sensitivity to men’s masculine identities beyond addressing erectile dysfunction.

In alignment with these endeavors, a 2018 study systematically reviewed interventions for prostate cancer survivors, aligning with American Cancer Society (ACS) and American Society of Clinical Oncology (ASCO) guidelines. This review found that both exercise and psychosocial interventions were effective in enhancing survivorship outcomes, addressing health promotion, physical well-being, and psychosocial challenges. However, it also highlighted gaps in existing studies and the lack of diversity in participant representation, emphasizing the necessity for targeted efforts to improve prostate cancer survivorship care [35].

Dunn et al. (2020) aimed to develop contemporary and inclusive prostate cancer survivorship guidelines for Australia, engaging a 47-member expert panel, including leaders from various clinical and community groups and diverse consumers [35]. This study identified six key descriptors for men’s current prostate cancer survivorship experience and 26 survivorship elements within six domains: health promotion and advocacy, shared management, vigilance, personal agency, care coordination, and evidence-based survivorship interventions. Although consensus was high regarding the essential nature of these domains, feasibility ratings varied. The study generated seven priorities for immediate action, offering valuable guidance for policymakers, clinicians, and the community to enhance prostate cancer survivorship outcomes comprehensively. Their results suggested the development of a survivorship framework prominently featuring “personal agency” as a central core element, recognizing the importance of empowering individuals to actively participate in their prostate cancer survivorship experience and make informed choices about their care and well-being [35].

Expanding on this research, a 2021 study conducted a systematic review of 22 randomized clinical trials, evaluating the effectiveness of psychological interventions in addressing depression, anxiety, and distress in prostate cancer patients [11]. The findings consistently demonstrated significant improvements in psychological aspects among patients receiving these interventions compared to those receiving standard care. Importantly, these positive effects remained consistent, even when specific assessment tools were used or when the study focused on patients with localized prostate cancer. Furthermore, interventions that combined cognitive and education-based approaches appeared to yield greater improvements in psychological parameters.

Mundle et al. (2021) emphasized the potential of psychological interventions to enhance the well-being of prostate cancer patients and called for further research to explore their impact on long-term clinical outcomes [11]. Additionally, a 2021 commentary stressed the need to discard the perception of psychological interventions as less significant than medical treatments for prostate cancer patients. It highlighted the interconnection between mental and physical health and criticized the limited attention given to psychosocial interventions, despite their recognized importance over a decade ago [31]. This commentary referenced the study by Mundle et al. (2021), which reinforced the effectiveness of psychological interventions in improving the psychological well-being of prostate cancer patients [31]. It underscored the importance of precise diagnosis labeling, clear intervention definitions, understanding mechanisms, and considering individual and cultural factors in treatment effectiveness. Overall, it advocated for rigorous research to enhance evidence-based practices in this field [31].

While cognitive-behavioral, educational, and peer support interventions have demonstrated associations with improved emotional well-being and health-related quality of life outcomes in men with localized prostate cancer, their integration into medical and survivorship care standards has remained limited. This limitation can be attributed to various factors, including inadequate evidence-based data, suboptimal methodology, small sample sizes, low-quality reporting, high rates of loss to follow-up, cost considerations, and limited diversity in the studied populations [11,31,33,34,35]. However, these reviews underscore the pressing need for targeted research interventions designed to address the multifaceted needs of patients [18,19,20,21,36]. Furthermore, robust assessments of intervention effectiveness and quality are imperative. To effectively address the present and future impact of prostate cancer on patients, their families, and communities, it is imperative to integrate these interventions into prostate cancer survivorship care plans [11,29,30,31].

This study aims to explore the perceptions of patients regarding a 28-day online Prostate Cancer-Patient Empowerment Program (PC-PEP). The program, developed through active patient engagement, focuses on patient education, activation, and well-being enhancement. Prior research has demonstrated its efficacy in reducing mental distress and improving physical fitness [29,30]. Through qualitative and quantitative assessments, we aim to gain insights into the perceived value of the program and its components, optimal delivery timing, program duration, and facilitators and barriers to adherence.

## 2. Materials and Methods

A qualitative study using a conventional content analysis approach was conducted as part of a Phase 2 feasibility study described elsewhere [30]. This type of qualitative analysis is appropriate for descriptors of a phenomenon when existing research literature on these issues is limited [37]. Thirty men who presented with a history of non-metastatic prostate cancer (Median age = 67; range 47–88 years old), spoke English, had access to email and a cell phone, that could receive text messages, and were able to travel three times to Halifax, Nova Scotia, Canada, were recruited and participated in the study between 12 January–10 February 2019. The patients learned about the PC-PEP study through posters placed in Urology and Radiation Oncology Clinics and Prostate Cancer Support Groups throughout Nova Scotia. Three focus group semi-structured interviews were conducted three days after program completion. Each focus group consisted of 10 men who were interviewed together and took approximately 45 min to complete. The quantitative program ratings/assessments were completed before the intervention after the initial PC-PEP training session and post-intervention, before the qualitative focus group interviews were rolled out. The quantitative ratings were administered by paper and pencil individually to each participant in the study and took about 5 min to complete. The interviews were conducted by two of the study’s co-authors face-to-face, in a private room, at the Dalhousie University’s gym, and were audio taped. The qualitative and quantitative questions from the structured part of the focused group interviews are described below. During the interviews, the patients were encouraged to elaborate and provide more information on their answers. All procedures followed were in accordance with the ethical standards of the responsible committee for human experimentation (institutional and national) and with the Helsinki Declaration of 1975, as revised in 2000. Informed consent was obtained from all participants in the PC-PEP study. The project (1021455) was approved by the Research Ethics Board of the Nova Scotia Health Authority, in Halifax, Nova Scotia.

### 2.1. The PC-PEP 28-Day Intervention

The 28-day PC-PEP Phase 2 study content, structure, administration, methodology and quantitative primary outcomes results are described elsewhere [30]. In brief, the PC-PEP is a comprehensive 28-day home-based program. It includes daily educational content and activities for forming new healthy habits. These activities encompass sleep, stress reduction techniques such as meditation, deep breathing, and recalling positive emotions. Additionally, the program offers daily exercise routines, including bodyweight and elastic resistance band (provided to patients for free to keep) strength exercises twice a week, aerobic exercises five times a week, and progressive pelvic floor exercises (3 times a day prompted by text alerts) with weekly video guidance. Dietary recommendations align with Canada’s Healthy Eating guidelines (https://food-guide.canada.ca/en/healthy-eating-recommendations/ (accessed on 20 September 2023)) and are delivered through daily emails. The program also provides education on intimacy and connection through video modules that encourage authentic communication, vulnerability, and compassion. To foster social support, participants can opt to be paired with two “buddies” undergoing similar treatment for their diagnosis. The participants attended training sessions on all aspects of the program prior to starting the program, then they received 28 daily messages (3–5 min long) outlining the education and daily activities for the day, and video resources guiding the daily activities. Every day participants were asked to engage in a total of 70 min of specific activities: 30 min of aerobic or strength exercises, 24 min (broken into 8 min sessions: morning, afternoon, and evening) of pelvic floor exercises, and 10 min of self-induced positive emotions through meditation and slow breathing.

### 2.2. Quantitative and Qualitative Evaluations

The quantitative questions of the structured part of the focus group interviews assessing the value, strengths and limitations of the program were devised by three of the co-authors with input from patients engaged in the research from the local prostate cancer support group during a conference pre-dating the feasibility study, who were not participants in the study. They were administered via paper and pencil questionnaires pre- and post-PC-PEP program delivery. Specifically, after the end of the training session day on 11 January 2019 (which explained and demonstrated the various components of the program to patients before the program began) patients were asked to rate their interest in the program overall (0, not interested at all; 10, extremely interested), their interest in each of the components of the program (pelvic floor, strength, diet, meditation and intimacy and connection techniques to help with problems associated with erectile dysfunction, and social support), their perceived quality of the PC-PEP training provided to patients, overall (0, not helpful at all; 10, extremely helpful), and their perceived competence of the research team who conducted the physical assessments and delivered the training for the program (0, not competent at all; 10, extremely competent). Three days after the end of the intervention, patients were asked to rate their interest in the program overall; rate how important/beneficial PC-PEP would be, in their opinion, if it were provided to patients from day one of diagnosis; the usefulness of the program to them personally; the usefulness of each of the program’s components; the competency of the research team; and their likelihood to recommend the PC-PEP program to men who have been diagnosed with prostate cancer on a Likert scale ranging from 0 to 10 (similar to pre- intervention assessments).

The in-depth, semi-structured focus group interviews were conducted with all patients, by two of the co-investigators (GI and RDHR) three days after the 28-day intervention was completed. The qualitative questions expanded on the post-intervention paper and pencil assessment. The participants were asked: “Please describe your experience with the PC-PEP program and what changes if any you noticed in your daily living habits? What aspects of the programs did you find facilitated your engagement and compliance with the program? How useful did you find the program, and its individual components of the program: daily videos from Drs. GI and RDHR reviewed the program requirements for the day, exercise videos of various strength levels using elastic bands, Kegel’s weekly videos, meditation and breathing videos and instructions, relationships and cancer education, intimacy and connection techniques to help with problems associated with erectile dysfunction teaching videos, relationships dietary recommendations, buddy system, Kegel text reminder, weekly compliance surveys, testing sessions prior to and at the end of the program? What aspects of the program or outside the program were challenging and might have prevented you from keeping up with the program and its requirements? In your view, what aspects of the program may require further development or improvement for the program to be more easily adopted by patients? From your point of view would PC-PEP be a valuable resource for a patient’s survivorship care from day one of diagnosis? Which of your prostate cancer survivorship needs did the program address or not address, from your perspective? What barriers do you think prevented you from benefiting from the program or what aspects did you perceive to not be helpful in your engagement with the program and its successful completion? What facilitated your engagement with the program and its successful completion? Other comments or feedback that you may wish to provide with regard to your experience with the program”. The average focus group interview length was 45 min for each of the three groups. The voluntary nature of participation and assurance regarding privacy and confidentiality were emphasized before each interview.

### 2.3. Data Analysis

All focus group interviews were audiotaped, and their content was transcribed verbatim, and reviewed by members of the research team (GI, HR, CM, RDHR) to identify major themes. The primary analysis team consisted of three co-authors (GI, HR and CM) who were experienced in qualitative research. The interviews were transcribed verbatim and analyzed within 12 months of data collection. During data analysis, three co-authors (GI, CM, HR) read the data word by word several times and identified emerging themes. Grounded theory methodology and content analysis were used in combination. With the analysis of the data, patterns, themes, and concepts were identified that emerge from the text. At this point, we decided to apply content analysis techniques to systematically categorize and summarize the explicit content within those emerging themes. This provided a structured way to present the data and enhance the rigor of the analysis.

The focus group text was divided into meaningful concepts known as meaning units (MU) coded based on similar features to create broad conceptual categories, and further classified into specific themes and sub-themes [38]. The authors focused on the recorded words and phrases used repeatedly by the patients and highlighted the areas that captured key meaning units expressed by the patients. The codes came straight from the data in the first level of coding. Words, phrases, or sections were noted and analyzed in context both within and between documents. As the next step, the multiple codes were grouped based on their content and shared ideas, which led to the creation of categories [37]. After identification of the categories, they were relabeled and defined one by one, while each was illustrated to support quotes. A constant comparison method was employed throughout each step of the coding process such that every time a MU was identified, it was automatically compared to the ones previously identified to determine distinct from similar classifications [39]. Any differences of opinion about the meaning or classification of meaning units in the transcripts were discussed and resolved collaboratively between the raters (GI, HR, CM) until at least 95% agreement was reached [39]. The results were then shared with another co-author (RDHR) for any further suggestions. At this point, the data from the interviews demonstrated saturation since data had become repetitive and nothing new appeared when coding data from the last interview when results were compared to suggestions from the fourth co-author (RDHR). To achieve the trustworthiness of the data, Lincoln and Guba’s credibility, transferability, dependability, and confirmability were used [40]. Prolonged engagement, peer debriefing, time triangulation, and member checking were employed to enhance the credibility of this study. The results content is presented, described, and summarized in a structured manner. Combining grounded theory and content analysis provides a more comprehensive understanding of the data and enhances the robustness of qualitative research findings. The agreement between each pair of two raters was very high (GI and HR, Cohen’s Kappa = 0.99; GI and CM, Cohen’s Kappa = 0.99, CM and HR, Cohen’s Kappa = 0.98).

Means and standard deviations were used to describe the pre- and post-ratings of the program. All 30 patients completed the 28-day program and all its quantitative and qualitative assessments, in their entirety. Sample characteristics have been published elsewhere [30].

## 3. Results

### 3.1. Qualitative Analysis

A content analysis of the focus groups revealed three main themes: (1) facilitating factors and perceived value of the PC-PEP components, (2) perceived appropriateness of the timing of access to the PC-PEP on the cancer journey, and (3) perceived challenges to engaging in the PC-PEP program during the prescribed time. Samples of quotes from the interviews illustrating the results below (for each section) are presented in the File S1.

#### 3.1.1. Facilitating Factors and Perceived Value of PC-PEP Components

All patients perceived the 28-day PC-PEP program to be extremely valuable and reported benefits from some or all components of the intervention. Patients stated that the on-line, home-based aspects of the program made the program easy to access and follow according to patients’ lifestyles, priorities, and daily routines. For patients who lived in remote areas, where there were no in-person prostate cancer support groups available, the “buddy” aspect of the program offered them access to a support group without the need to travel anywhere, and a “friendly accountability” to their keeping up with the various components of the program. The weekly compliance surveys were also perceived by patients to increase their accountability. Patients stated: “*at the back of your mind you got, oh well okay I’m going to be held accountable—I’m going to be held accountable, and yeah I should keep on doing this, but without [the weekly compliance survey] then I might just slip back*” (File S1). Patients stated that the contact with other men from the program, many of whom were survivors, helped alleviate feelings of loneliness, isolation, and provided support, especially the patients who were grieving their loss of sexual function following radical prostatectomy. The patient articulated how the buddy system helped them feel validated by someone who experienced the same type of treatment and accompanying side effects (File S1).

Most patients stated that they were living with a reduced quality of life because of some of the side effects associated with their treatment (e.g., loss of sexual function, sedentarism, depression, isolation), and they were struggling with overcoming these side effects on their own. Two-thirds of the patients stated that their treatment (e.g., surgery, radiation therapy) rendered them unable to have or maintain an erection, and experienced decreased or loss of libido. Patients stated that their loss of sexual function was accompanied by a decrease in the ability to meet their emotional needs such as feeling respected, loved, appreciated, feeling admired, feeling that their partner is proud of them, feeling attractive, and feeling that they are a priority and accepted for who they are. Among younger patients, the switch from being sexually active to losing sexual function following treatment was reported to have been psychologically traumatic and compared to “*going from 57 to 75 years old, overnight*” (File S1). Patient stated, “*My testosterone levels have dropped from 100% for someone at my age to 3%, overnight. So, emotionally I feel I’ve been much more fragile over the past couple of weeks*”. Through the PC-PEP program, patients reported an enhanced understanding of the practical ways in which diverse forms of intimacy (e.g., recreational, intellectual, emotional, self, physical, unconditional and others) can play in providing emotional support (e.g., communication of appreciation, value, respect, and acceptance) and overcoming feelings of isolation and disconnect. Patients noted that the program facilitated their understanding of identifying their preferred mode of communication and fostering awareness of the preferred communication styles (love languages) of those in their social circles. This awareness aided in initiating more profound connections and interactions. The encouragement to explore secure, suitable, mutually advantageous avenues for connecting with loved ones, which augmented emotional stability and bolstered relationship contentment, was viewed positively. Certain patients also highlighted the program’s positive influence on their proximate and long-distance relationships. They reported a heightened willingness to engage in vulnerability and genuine sharing during telephone conversations or face-to-face interactions with partners and distant relatives. One patient stated: “*It’s been so tremendously valuable to me, and I really hope that there are ways found to enable more men and their loved ones to benefit from this type of support program that will enable them to lead better lives. Because, you know, the good thing about prostate cancer is that [it has a] 95% 10-year survival rate. But there’s being alive and then there’s living… and I think there’s 95% of people that are alive—but I’m not sure what percentage of them are still really living. And if we can improve that percentage, I think that would be incredibly valuable*”. Another patient stated: “*When I started this program, I was struggling, and this program was a way for me to turn it around. […] This program is invaluable, and it should be for everybody*” (File S1).

The ability to have a social support network (“buddy system”) and the encouragement to cultivate connections (delivered by the program’s leads through daily email videos) was perceived as a facilitating factor. This facet of the program engendered a feeling of inclusion and concurrently enhanced mental well-being. Most patients not only acknowledged the advantages derived from the support provided by fellow participants who were also survivors, but they also appreciated the heightened depth of their relationships with partners and family members during the program’s duration. Three patients stated the program, however, had a small to no influence on their relationships (“*what we did was share in talking more, as a result of the program, but that’s it*”).

Patients who were in the middle of their treatment stated that having expert support and encouragement to exercise daily (from home, with equipment that was provided for free—elastic bands), to be encouraged to eat healthy foods and be told why this is important, to connect with others for social support, and connect more closely with loved ones, were essential elements to their perceived program’s success. Most patients highlighted that they lost weight and that they were very pleased with the impact the program had on their overall physical fitness. Patients also stated that the program gave them achievable goals and reignited their motivation to exercise which they had previously lost. All patients indicated the intent to continue to exercise after the program ended (File S1).

Patients stated that urinary incontinence was a common side effect of either their radical prostatectomy and/or radiation therapy and perceived this to have been a significant factor in their perceived decreased quality of life. Most patients found the Kegels component of the program highly valuable for improving their urinary function. Patients discussed how the specific Kegels education in PC-PEP differed from other previous Kegels education they received. Twelve patients stated that they were handed a pamphlet in the hospital that described Kegel exercises with no additional instructions, while eight patients stated that they were seen by a pelvic floor nurse on a weekly basis for several weeks or paid for a psychotherapy nurse to provide them with instructions on how to do the exercises correctly). During the PC-PEP program patients stated that they were not only instructed on how to do the exercises through explanatory and demonstrative videos, but they had a meeting with a nurse Kegels specialist right at the start of the program and received daily text alerts via their phone three times a day instructing them to do the 8 min of Kegels prescribed for that week. Patients attested that in their view this difference contributed to an improvement in their urinary control which they noted. Some participants stated that they went from using three urinary pads a day to being 95% dry at the end of the 28-day program. Patients stated that the change in the Kegels routines from one week to the other, made this a progressive training program, and they perceived this aspect of PC-PEP to be a helpful, and effective feature of the program. However, not every patient had urinary issues; when asked if they complied with this aspect of the program even if they did not have urinary problems, most participants said they did, adding that they perceived this to be a helpful skill to have for future age-related urinary problems (File S1).

While acknowledging the significance of exercise, Kegels, and other program components for enhancing post-prostate cancer treatment quality of life, most patients admitted lesser awareness concerning aspects such as meditation, meeting emotional and intimacy needs, and their impact on perceived well-being. Numerous patients expressed a gap in their understanding of practical strategies to effectively manage both intimate and non-intimate relationships during their cancer journey, along with the potential influence of these aspects on their holistic well-being. These individuals acknowledged that the program’s emphasis on social support and pursuit of diverse forms of intimacy (recreational, emotional, physical, intellectual) facilitated their progression toward acceptance, healing, and alternative avenues for fulfilling psychological and emotional closeness (File S1).

The majority of patients were initially unfamiliar with the beneficial impact of meditation and slow deep breathing on stress reduction. Despite finding the concept of meditation somewhat bothersome initially, most participants noted that the daily prompts for meditation and breathing exercises contributed to enhanced relaxation throughout their day and heightened awareness of their emotional state over the course of the week. Notably, the integration of technology and diverse communication channels (app notifications, daily emails, text messages, videos) for program delivery proved highly effective in both motivating patients and maintaining their adherence to the program (File S1).

In general, patients expressed that the program reinstated their confidence in adopting healthier lifestyle habits and empowered them to actively address the changes and challenges they encountered both physically and psychologically after cancer treatment. They conveyed an enhanced sense of self-efficacy, feeling capable of positively impacting their own health and well-being. The program activated their autonomy, allowing them to assert control over their role in supporting their health and lifestyle. Describing the PC-PEP program as a “gestalt”, patients emphasized that its collective impact exceeded the sum of its individual components. In essence, the program was more than its parts, combining knowledge, motivation, accountability, and support to benefit and empower participants (File S1).

#### 3.1.2. Perceived Appropriateness of the Timing and Duration of Access to PC-PEP on the Cancer Journey

Although all patients acknowledged the significant benefits of the PC-PEP program, they also expressed that they would have likely gained even more from it had it been offered earlier in their survivorship journey, even as early as their initial diagnosis. This sentiment was particularly pronounced among patients currently undergoing treatment. In hindsight, patients recognized that the program could have provided invaluable consolation during the challenges of treatment by directing their attention to controllable aspects such as lifestyle choices. They believed it could have assisted in preparing for treatment-related side effects and even influenced treatment decisions, potentially reducing apprehension around options such as active surveillance.

The majority of patients found the 28-day duration of the PC-PEP program suitable; however, they proposed two potential enhancements: the introduction of follow-up sessions (e.g., monthly or ongoing) or an extension of the program’s duration (e.g., to 3 or 6 months) (File S1). Even those who deemed the original 28-day timeframe appropriate underscored the value of follow-up mechanisms to sustain engagement and continuity. In general, all patients stressed the importance of ongoing program access and regular follow-ups, as these elements would aid in preserving the newly developed habits by maintaining motivation and accountability over the long term. Patients also commended the ease of implementing the program within the initial 28-day period.

#### 3.1.3. Perceived Challenges to Adhering to the PC-PEP Program during the Prescribed Time

In general, the majority of patients encountered few obstacles during their engagement with the PC-PEP program within the stipulated 28-day timeframe. Among the 30 participants, five individuals reported specific challenges. These included concerns about the time commitment for daily program activities (involving 70 min of prescribed engagement daily, which some likened to a “cancer survivorship boot camp”), work-related scheduling conflicts, personal aversion to meditation and slow breathing exercises, and environmental limitations such as winter weather impacting outdoor aerobic activities. While the program catered to both partnered and unpartnered men, those who were single or lacked a willing partner for the intimacy-focused activities expressed regret at not being able to partake in partner-oriented exercises. One patient cited his partner’s advanced age as a factor preventing deep connection and intimacy engagement. Additionally, a subset of patients experiencing relationship difficulties noted that the intimacy education aspect of the program illuminated existing challenges in their partnerships, leading to a constructive examination of relational dynamics. While this was deemed positive, it was acknowledged that this aspect might present challenges to future participants. Some patients found the social connection facet of the program somewhat demanding, particularly as age-related isolation could impact their ability to engage fully. For instance, reaching out to distant acquaintances as prescribed on Sundays became more complex when confronted with the reality of friends’ passing or drifting apart. Despite these hurdles, all patients affirmed that their quality of life underwent positive transformations upon completing the program, underscoring the overall benefits they derived, even in the face of challenges.

The daily dietary prompts were deemed beneficial, though patients suggested enhancing the dietary element by including a pamphlet, cookbook, or cooking videos alongside the daily video reminders. Additionally, a couple of participants shared that engaging in the buddy and mentor features of the program occasionally led to social awkwardness when discussing program-related interactions with other men. Nonetheless, these instances were generally seen as positive experiences. One patient elaborated that discomfort in connecting with a buddy occasionally stemmed from difficulty determining the optimal timing for contact, resulting in avoidance. For instance, they noted that reaching out sometimes coincided with the buddy’s unavailability or preoccupation, causing hesitation. Despite these observations, most patients actively participated in the buddy component, built meaningful relationships and found the experience very rewarding (File S1).

### 3.2. Quantitative Analysis

Table 1 outlines the characteristics of the sample. No attrition was observed. The participants’ ages ranged from 56 to 83 years, with an average age of 68.93 years. The majority of patients were White/Caucasian (93%), had a university education (67%), were in a relationship (100%), either retired or unemployed (70%), had a household income over 80,000 CAD (73%), were diagnosed 25 months before the study began (73%), and had radical prostatectomy (47%), radiation therapy (with our without hormone therapy) (20%), or were on active surveillance (13%) and were not very active to moderately active both before and after the intervention. The majority of participants received enough information from the hospital about their diagnosis (83%) and the available types of treatment (63%) and reported being satisfied or extremely satisfied with the education materials received from the hospital at baseline (46%). A decrease in treatment regret was observed, with 17% reporting regret pre-intervention and 10% post-intervention. The percentage of participants attending support groups increased from 33% pre-intervention to 43% post-intervention.

Table 2 and Table 3 outline patients’ evaluation of the program pre- and post- intervention, respectively. Overall, patients found the program to be very useful when introduced (before the 28 days trial) and post-intervention with average evaluations for the components of the program ranging from M = 8.55 to M = 9.73 (out of 10), and M = 7.46 to M = 9.89 (out of 10), respectively.

The lowest-rated aspect of the PC-PEP after the training session and before the program began was the meditation component (M = 8.55, SD = 1.99) while the highest-rated was the pelvic floor training component (M = 9.66, SD = 0.67). After the PC-PEP was completed, the lowest-rated aspect of the program was the meditation component (M = 7.46, SD = 2.05) while the highest was the daily video and email messages prescribing, describing, and demonstrating the various components of the program (M = 9.47, SD = 0.82). The competency of the leads of the PC-PEP was rated 9.73 (SD = 0.52) and 9.89 (Sd = 0.32), pre and post-intervention, respectively.

The program received a strong likelihood of recommendation from patients to their peers (mean = 9.79, SD = 0.42) and was rated as highly valuable, averaging 9 out of 10 (SD = 1.19), particularly if it were to be administered upon diagnosis. Patient interest in the program remained consistently high, with comparable levels observed from the pre-test (mean = 8.87, SD = 1.70) to the post-test (mean = 8.89, SD = 0.99).

## 4. Discussion

There is a paucity of research evaluating multifaceted health promotion lifestyle interventions for prostate cancer patients and survivors. This study aimed to evaluate patients’ feedback and perspectives on the overall PC-PEP and its components, through a content analysis of their semi-structured focus group interviews. Overall, patients had a positive experience participating in the 28-day PC-PEP program. These results provide context to the physical and mental health quality of life improvements previously reported and offer opportunities for program improvement as well as support in its integration into standard care for prostate cancer patients [29,30].

The first theme that emerged from the group semi-structured interviews, as well as the quantitative ratings, was an overall high perceived value of the PC-PEP program in meeting patients’ education and survivorship needs. Patients stated that the program filled a gap between the medical care they received in the hospital and their psychosocial survivorship needs that could be traced back to the time of diagnosis, which became more complex post-treatment due to treatment side effects. Indeed, research shows that prostate cancer patients report receiving inadequate information on strategies to address psychosocial needs cooccurring with oncological treatment, such as the need for social support, the adoption of healthier lifestyle habits related to sleep, physical fitness, eating, and stress around treatment and the possibility of cancer reoccurrence [32,36,41,42]. Patients reported feeling empowered, having greater agency and self-efficacy and confidence that they can better manage their treatment-related side-effects and improve their overall health. Patient agency was reflected in the patient’s ability and willingness to exercise their autonomy and actively participate in their healthcare, including reporting advocacy for their needs and preferences [35]. These reports are congruent with the mental and physical health improvements the men in the program had following completion of the program (28-day and 6-month versions), which we reported elsewhere [29,30]. These findings are comparable to findings from other studies that have evaluated supportive care interventions for prostate cancer patients leading to an improvement in self-efficacy and emphasize the importance of consumer-led and grassroots movements in prostate cancer, underscoring their pivotal role in shaping supportive care initiatives and promoting empowerment among patients [11,31,33,34,35,42,43,44,45].

Meaningful conclusions about the effect of the PC-PEP intervention, however, also require an understanding of its components and how they might have driven its effectiveness. Patients reported that they valued the multifaceted nature of the program and the “gestalt” it represented. This qualitative descriptive result was corroborated by patients’ quantitative mean ratings of their perceived value of the program, overall (M = 9.61, SD = 0.57), post-intervention. Interestingly, the mean rating for the program in its entirety was higher than the mean ratings for each of the individual components of the program (Table 2). This may suggest that the program was perceived as a good fit for the complex needs experienced by prostate cancer patients. The alignment between patients’ needs and the tailored solutions provided by PC-PEP can potentially be attributed to the intervention’s “bottom-up” development approach, underscored by patient engagement as its foundational principle. Indeed, to our knowledge, this is the first home-based comprehensive program of this complexity to be developed through patient engagement from the start, to provide daily education and prescription of healthy habit formation while holding patients accountable for their engagement in the program through weekly compliance surveys [29,30]. Research on patient engagement shows that when patients are engaged in the development of a behavioral intervention, they are more likely to adopt it [33,46]. Indeed, unhealthy living habits and behaviors account for a substantial combined burden on the life expectancy of Canadians and Americans [47,48]. Engaging and activating patients’ role in their care, and in developing healthy behavioral habits has been previously shown to improve the health of patients [49,50].

Patients reported that the PC-PEP program gave them agency and motivation to take better care of themselves and return to a happier way of living. However, although the group lost weight, “weight loss” was not discussed or emphasized during the program, instead the leads emphasized through daily emails, the importance of staying fit to feel good, support good health, and a good quality of life. Research has demonstrated several benefits of exercise for weight loss in prostate cancer patients. Engaging in regular physical activity as part of a comprehensive program can lead to weight loss, improved body composition, and overall better health outcomes [51,52]. Patients particularly highlighted their appreciation for the intimacy and connection education sessions of the program, and reported it made them feel more connected, and it improved their communication with loved ones. This was noted not only by patients who had a loss of sexual function due to active forms of treatment (e.g., surgery) but by all patients. These results are corroborated by other research showing that educational sessions focused on intimacy and connection strategies for single men and men who are in a relationship are helpful in promoting better quality of life and relationship satisfaction [43,44,53].

Patients further reported that the “buddy” aspect of the program helped them “keep each other accountable” and “helping each other” stay on schedule, which facilitated forming new healthy habits. These results are corroborated by research showing positive habit formation and sustained maintenance of healthy habits can be facilitated through social support [54,55,56]. Focus groups also revealed that participating patients endorsed the implementation of the PC-PEP program in the standard of care for prostate cancer patients from day one of diagnosis. All patients reported that implementing the program from day one of diagnosis, or before active treatment was scheduled or planned, would help reduce their stress and activate their role in their own health care that can work alongside the medical system to help promote a better quality of life. These results from our focus groups are not surprising. Research shows that the management of cancer care often fails to recognize the need for or delivering patient-centered conversations, education about treatment choices, their individual side effects, and their management, and in particular, their quality-of-life implications [57,58].

While focus group interviews revealed that patients perceived the PC-PEP intervention to be of an appropriate length of time (28 day), they suggested it be expanded to 3 or 6 months to allow their newly developed healthy habits to solidify. Patients perceived the program to be demanding (e.g., 70 min of activities daily) yet they still supported the idea of extending the duration of the program to 3 or 6 months, to maintain newly formed healthy habits. Indeed, one highly cited study on the duration needed for new healthy habit formation shows that it takes between 18 to 254 days (median 66 days) to form and maintain new healthy habits long-term and ongoing engagement to help facilitate subsequent automatic adherence is necessary [59]. Habit formation is a complex phenomenon that depends on the type of behavior intended to change, as well as individual and context-related factors [60]. The PC-PEP program has since been expanded to 6 months, with additional on-going (indefinitely) monthly videoconferences for check-ups, updates and support, and was tested in a Phase 3 randomized clinical trial showing mental and physical benefits at the end of the intervention (6 months later), with benefits lasting and continuing to improve at 12 months post-intervention [29].

Focus group interview results showed that the PC-PEP program was well received by all participants, with only a few patients perceiving some aspects of the program as challenging. The “boot camp” aspect of the program was perceived as challenging by some participants, but it was also perceived as highly successful and resulted in average weight loss for the group from pre- to post-intervention [30]. Previous studies involving breast cancer survivors showed that “boot camp” type programs (three to 5 weeks long) were successful at promoting healthy physical fitness habits although several barriers to long-term retention have been noted (e.g., physical injury, bad weather and competing events) [61,62]. We note that our results also point out to challenges some patients had with program adherence which include employment, weather, and personal circumstances (stated as a barrier to engaging in the “buddy” aspect of the program). A few participants found the vulnerable sharing between men, who were faced with the same disease, “embarrassing”, and noted that men tend not to speak with other men about intimate and personal details of living with the disease. These results suggest that the gender norms of masculinity introduced by society may be acting as a barrier for men seeking social support [63,64]. Indeed, men are much less likely to ask for professional help for their mental health concerns compared to women [64]. Recent research shows that strategies relating to a malleable interpretation of gender roles and perceived masculinity may be a particularly effective way to promote vulnerability and authentic communication and improve social support, which in return has been shown to have a beneficial impact on psychological well-being [65]. Furthermore, studies have shown that patients’ engagement and their level of activation in their care, self-management, and flexible self-perception can predict outcomes over several years and that when the level of engagement and activation change, psychosocial and health-related (including health care utilization) outcomes also improve [49,66].

Participants found the prescribed strength, aerobic, and Kegel exercises beneficial for their overall perceived quality of life, in line with literature demonstrating exercise’s positive impact on prostate cancer survivors’ quality of life [51,52,67,68]. Many patients also valued the PC-PEP’s dietary recommendations and meditation/stress reduction breathing techniques which have been shown to positively impact psychological well-being and contribute to enhanced coping strategies in cancer survivors [69,70,71,72]. Similarly, the social support component received positive feedback from most participants. Research has shown that social connection improves the quality of life and mental health outcomes for prostate cancer survivors [54,55]. Lower social support levels may correlate with reduced quality of life, suggesting that interventions connecting patients with their social circle can be beneficial [55]. Overall, patients embraced the individual program components.

Another factor that may have played a significant role in program adherence [30] and merits discussion is the PC-PEP leads’ expertise. PC-PEP’s daily videos were led and delivered by two of its co-authors, a scientist in prostate cancer quality of life research (GI) and a prostate cancer radiation oncologist (RR). For the delivery of PC-PEP and daily video creation, the leads invested time, effort, and attention to finding effective ways to support patient self-management, and delineated, in their video presentations, the role of the medical system from the role patients can play as active participants in their care (e.g., exercising, eating healthier, staying connected with loved ones and seeking social support). Although outside of a clinical setting, the program leads adopted a “coach” role, instructing patients on patient agency, healthy living habits and the science behind healthy living practices [49]. Making explicit the delineation between the role of the medical system and the role of the patient in self-care and agency is not a strategy that is typically included and made explicit in discussions of best practices but may be an important aspect of patient education and clinical training [35,49,50,66,73]. Indeed, for patient mental and health-related outcomes to improve, patients must do their part, following through on prescribed treatments, and making recommended lifestyle changes [33,34,35,50,66,73,74]. In recognition of the key role that patients play in influencing mental and physical health outcomes, policy makers have made patient engagement a priority, including embedding approaches for increasing patient engagement in the Patient Protection and Affordable Care Act [33,34,35,74,75]. PC-PEP daily video messages, which conveyed role delineation and encouraged patients’ activation in their care, were perceived by patients as extremely helpful, supportive, and empowering and were rated on average 9.15 out of 10. The competency of the program’s lead was also perceived as high, 9.89/10. Together they may have contributed to the PC-PEP intervention’s success and the program’s high rates of compliance whether over the shorter duration of 28 days or the extended period of 6 months [32,33]. Indeed, health recommendations provided to patients by a clinician or health care professional can action positive changes in overcoming unhealthy behaviors especially when patients’ ownership over their own health is emphasized when a sense of partnership in care between clinician and patient is conveyed, when small steps toward the desired changes are identified, and a sense of genuine personal care for patients is communicated [73]. All these steps were reflected in the delivery of the PC-PEP program. Actioning behavioral changes in patients is, however, a complex process affected by many factors including and not limited to individual characteristics, motivation, and several other contextual factors [56,66]. To our knowledge, there are no current multifaceted programs that prescribe daily activities led by a clinician and scientist that require a daily commitment to each of the aspects of the program to which the results of our study could be compared. More research is warranted to better understand the potential causal chain that links better outcomes with patients’ perceived higher value for multifaceted (over single component) interventions that address their needs, as well as the potential mediating role of patient, scientist, and clinician engagement and activation in the delivery of such interventions. Results here suggest that clinical training in patient empowerment, activation, agency, and self-management to help support patients on their cancer journey and encourage their participation in their care and survivorship may be warranted [35,74,75].

### Limitations

This qualitative study is not without limitations. The utility of qualitative data is strongly linked to the effectiveness of the researcher’s interviewing techniques. Although every attempt was made to keep interviews standardized and semi-structured not all patients in the focus group answered every question posed during the focus groups, particularly if another patient articulated what they intended to say. This may have introduced systematic variations in the type and detail of information shared. Second, the study’s reliance on focus group data from a convenience sample of 30 men who voluntarily participated in the intervention introduces a potential bias, as these participants may be more inclined to view the intervention favorably. This limitation underscores the challenge of generalizing findings from this specific sample, particularly when considering the influence of contextual factors, such as masculine values, which must be considered when interpreting the results. Another study limitation is that the focus group interviews were conducted by the program’s leads. This might have led participants to a positive bias in their responses. We note, however, that the quantitative assessments (completed independently prior to the focus group interviews were completed remotely and independently by each participant) highly mirror the qualitative responses provided during the focus group interviews. Nonetheless, future studies should attempt to replicate the qualitative assessments for the PC-PEP with independent interviewers. Two follow-up studies by our team are underway attempting to qualitatively evaluate the effectiveness of Phase 3 RCT and Phase 4 multisite implementation trials, testing the effectiveness of a 6-month PC-PEP program with interviews performed by independent interviewers [32,76].

Given the voluntary nature of the study, not all patients’ viewpoints may have been represented. While our sample was sufficient to attain saturation, future work should attempt to gain a greater representation of diverse subpopulations of patients and survivors during their cancer journey.

Despite these limitations, this study has many strengths. The concurrent use of quantitative ratings and focus group interviews allowed for a comprehensive assessment of participants’ perceptions regarding the program’s value and integration into standard care for prostate cancer patients. The congruence between the qualitative and quantitative findings enriched the evaluation from the patients’ viewpoint. While exercise, pelvic floor training, intimacy, connection, stress reduction, and social support all contribute to enhanced quality of life for prostate cancer patients and survivors, no other intervention has integrated these facets into a singular program, systematically educating and empowering patients, maintaining accountability through weekly compliance surveys, and being guided by qualified professionals. The PC-PEP program effectively aimed to address common unmet needs reported by patients during their prostate cancer journey, thereby enhancing their quality of life. Findings from both qualitative and quantitative assessments, both in this study and others, have demonstrated the feasibility, safety, and positive impact on mental and physical health [29,30]. A longer variant of the program spanning six months, recommended by patients and recently piloted, incorporates ongoing live monthly video conferences with breakout sessions and introduces a mentoring system alongside the existing “buddy” system [29]. Patient education, led by a scientist and clinician with a collaborative and patient-centered approach, has the potential to improve patient well-being, mental and physical outcomes, and potentially reduce medical costs related to prostate cancer survivorship. The expansion of the PC-PEP program across Canada and globally, endorsed by enthusiastic patients, signifies its robust impact and promising potential [76]. This program’s integration into the standard care for prostate cancer patients in Nova Scotia is currently underway [29,76].

## 5. Conclusions

While cutting-edge medical procedures and innovative therapies are essential in addressing prostate cancer, they often fall short of achieving optimal outcomes when used in isolation. Patient agency, education, and empowerment play pivotal roles in the survivorship journey, fostering enhanced self-efficacy, autonomy, and preparedness to manage treatment side effects, thus significantly contributing to the overall quality of life [29,30,33,34,35,50,66,75]. Notably, many prostate cancer patients frequently report inadequate education from their physicians regarding the management of treatment-related side effects [54]. Therefore, patients’ active engagement in their care, combined with their willingness to participate in care decisions and processes, emerges as crucial components of comprehensive patient care throughout the prostate cancer journey.

Contemporary research increasingly focuses on developing comprehensive approaches to support men diagnosed with prostate cancer [11,29,30,31,33,34]. These efforts aim to assess the impact of interventions that address the multifaceted needs of patients, with the ultimate goal of enhancing mental and physical well-being, ultimately improving overall quality of life [11,29,30,31,33,34]. The current work not only enriches the context provided by quantitative approaches but also delves into patients’ perspectives on the PC-PEP program. This qualitative exploration offers invaluable insights into its perceived value, as well as the identified barriers and facilitators. Through this qualitative lens, we capture the real-world experiences, challenges, and triumphs of patients, which may be overlooked in quantitative studies. Collectively, these insights provide a deeper understanding of patients’ lived experiences and viewpoints within the realm of survivorship care for prostate cancer.

This information is crucial for understanding how such interventions are received by patients and how they can effectively address unmet needs in survivorship care. Notably, patients’ recommendations regarding the integration of the program into standard care represent practical and actionable guidance. These insights can inform healthcare providers and policymakers when considering the incorporation of similar patient empowerment and education programs as routine components of care for prostate cancer survivors.

The qualitative findings also shed light on the intricacies of survivorship needs, extending beyond medical treatment to encompass psychosocial, lifestyle, and emotional aspects. These facets are often inadequately addressed within standard clinical care, emphasizing the importance of holistic survivorship programs. Additionally, the study underscores the pivotal role of patient engagement and empowerment in improving outcomes, aligning with the broader healthcare trend toward patient-centered care. Finally, the study’s emphasis on involving patients from the outset of program development carries significant implications. It suggests that such patient-centered approaches can lead to more effective, tailored, and patient-focused interventions. This insight has the potential to guide future research and program development endeavors.

In summary, the findings presented here underscore the value of collaborative efforts between physicians and scientists in implementing patient education and empowerment initiatives, particularly during the challenging phases of the cancer journey. Establishing patients as active partners in their care remains of paramount importance. Clinicians should be encouraged to cultivate robust skills in patient activation, education, and empowerment, alongside strategies for shared decision-making, thereby fostering meaningful patient engagement.

## Figures and Tables

**Table 1:** Sample characteristics at baseline of 30 men who participated in the 28-day PC-PEP Phase 2 study, from the Maritimes, Canada.

Sex	Male: n = 30 (100%), Female n = 0 (0%)
Age	Mean: 68.93 years, Range: 56–83 years old
Ethnicity	White/Caucasian: n = 28 (93%)
Education	University: n = 20 (67%)
Relationship status	In a relationship: n = 30 (100%)
Employment status	Retired or unemployed: n = 21 (70%)
Household income	Less than 80 K/year: n = 8 (27%)
Time between diagnosis and baselinesurvey	More than 25 months: n = 22 (73%)
Treatment modality	Active surveillance: n = 4 (13%) Radical prostatectomy: n = 14 (47%) Radiation (beam, brachy or seed) +/− hormone: n = 6 (20%) Radical prostatectomy, radiation and hormones: n = 4 (13%) Androgen Depravation Therapy: n = 2 (7%)
Current physical activity level	Pre-Intervention: Not very active (<30 min/week) to moderately active (30-150 min/week): n = 24 (80%) Post-Intervention: Not very active (<30 min/week) to moderately active (30–150 min/week): n = 19 (63%)
Current support group attendance	Pre-Intervention: Yes: n = 10 (33%) Post-Intervention: Yes: n = 13 (43%)
Treatment regret	Pre-Intervention: Yes: n = 5 (17%) Post-Intervention: Yes: n = 3 (10%)
Reports of sufficient information received from the hospital at baseline with regards to	Diagnosis: n = 25 (83%) Available types of treatment: n = 19 (63%) Best diet: n = 9 (30%) Best form of exercise: n = 8 (27%) Impact on relationship with partner/sexual life: n = 14 (47%) Participants’ specific treatment type: n = 13 (43%) Other side effects/consequences on quality of life: n = 3 (10%)
Patient rated satisfaction with the education materials received from the hospital at baseline	Not satisfied at all: n = 6 (20%) Somewhat satisfied: n = 5 (17%) Satisfied: n = 10 (33%) Mostly satisfied: n = 5 (17%) Extremely satisfied: n = 4 (13%)

**Table 2:** Pre-PC-PEP evaluation (out of 10) after the half-day PC-PEP Training session/presentation describing all aspects of the program before the program began, as rated by participating program patients from Halifax, Canada, n = 30.

Perceived interest in PC-PEP program	M = 8.87, SD = 1.70
Perceived importance of the PC-PEP program	M = 9.43, SD = 1.01
Perceived usefulness of the Science Behind the PC-PEP program session/presentation	M = 9.47, SD = 0.82
Perceived usefulness of the PC-PEP Pelvic Floor session/presentation	M = 9.66, SD = 0.67
Perceived usefulness of the PC-PEP Meditation session/presentation	M = 8.55, SD = 1.99
Perceived usefulness of the PC_PEP Physical Activity session/presentation	M = 9.20, SD = 1.03
Perceived usefulness of the PC-PEP Connection and Intimacy session/presentation	M = 8.80, SD = 1.38
Perceived usefulness of the entire half-day PC-PEP Training session/presentations	M = 9.47, SD = 0.82
Perceived competence of PC-PEP Team during the half-day PC-PEP Training session	M = 9.73, SD = 0.52

M—mean; SD—standard deviation.

**Table 3:** Post-PC-PEP program evaluation (out of 10) after the 28-day program held between 12 January to 10 February 2019, as rated by participating patients, from Halifax, Canada, n = 30.

Perceived interest in the PC-PEP program after completion of the PC-PEP program	M = 8.89, SD = 0.99
Perceived importance of delivering the PC-PEP to newly diagnosed patients from day 1 of diagnosis	M = 8.54, SD = 1.20
Perceived importance of delivering the PC-PEP program from day 1 of diagnosis to the participant	M = 9.61, SD = 0.57
Perceived overall usefulness of the PC-PEP program	M = 9.00, SD =1.19
Perceived usefulness of the PC-PEP Pelvic Floor aspect of the program	M = 8.75, SD = 1.35
Perceived usefulness of the PC-PEP Meditation aspect of the program	M = 7.46, SD = 2.05
Perceived usefulness of the PC-PEP Physical activity aspect of the program	M = 8.75, SD = 1.35
Perceived usefulness of the PC-PEP Connection and Intimacy aspect of the program	M = 8.00, SD = 1.25
Perceived usefulness of the PC-PEP daily videos and email message of the program	M = 9.15, SD = 1.75
Perceived competence of the PC-PEP program leads	M = 9.89, SD = 0.32
Likelihood to recommend the PC-PEP to other men diagnosed with prostate cancer	M = 9.79, SD = 0.42

M—mean; SD—standard deviation.

## Data Availability

Data from this study are available to re-searchers through a data access process in compliance with patient privacy and protection research act (NSHA Research Ethics Board).

## Supplementary Materials

The following supporting information can be downloaded at: https://www.mdpi.com/article/10.3390/curroncol30090626/s1. File S1: Interview Excerpts.
