# Supplementary material for: Assessing the Efficacy of a 28-Day Comprehensive Online Prostate Cancer Patient Empowerment Program (PC-PEP) in Facilitating Engagement of Prostate Cancer Patients in Their Survivorship Care: A Qualitative Study
Source: Curr Oncol. 2023 Sep 21;30(9):8633–52. doi: 10.3390/curroncol30090626 (PMC10528560; doi:10.3390/curroncol30090626)
Supplement: Supplementary file 1 [file curroncol-30-00626-s001.zip › curroncol-2590946-supplementary.pdf]

Supplement Materials

Overall value of the program.

"I'm down in the Valley, there is no support group down here. The "Buddy" system of the program gave me someone to talk to that had the same conditions I had and it gave a friendly accountability. […] And I never would have had that if it wasn't for this program. And I don't have to travel anywhere to get access to all this."

"It's been so tremendously valuable to me and I really hope that there are ways found to enable more men and their loved ones to benefit from this type of support program that will enable them to lead better lives. Because, you know, the good thing about prostate cancer is that [it has a] 95% 10-year survival rate. But there's being alive and then there's living…and I think there's 95% of people that are alive – but I'm not sure what percentage of them are still really living. And if we can improve that percentage, I think that would be incredibly valuable."

"… after my surgery I was depressed, started not eating as well as I had before, I wasn't exercising, basically I became a couch potato, so the surgery had a profound effect on me, and the loss of sexual function had a profound effect on me. When I started this program, I was struggling, and this program was a way for me to turn it around. […] This program is invaluable, and it should be for everybody."

"I am right in the midst of my treatment right now, for prostate cancer, I had surgery, now I am in hormonal treatment, getting prepared for radiation treatment down the road so I wanted to be as prepared as I could be for that. So, the idea of having expert support and encouragement, and challenge, I thought was something I thought I would need. I've learned that it is important to work out a lot. It's been great being able to be part of a group with other men who have prostate cancer as well. Because, when I got my diagnosis initially, was kind of like a lonely thing. You very much feel that. How am I going to handle this? How is my wife and I going to handle this. And then to be able to come into a group of people and connect with some other men who've been going through it, who are down the road in terms of treatment, it's been very helpful."

"You know, the diet, exercise, all that I would have anticipated to be valuable. The meditation, I never would have, I wouldn't have told you up front, I wouldn't have said yeah that's going to be very useful".

"It's that sense that I can own it, I can take some charge of it, I'm not a leaf on the stream of life getting carried wherever it's going to take me. I can actually change that, and that's been incredibly valuable for me".

"…the Kegel exercises, the structured physical workouts … and I never would have thought of meditation … but it's truly all those aspects I think have come together to really improve my physical sense of well-being".

"…collectively the sum of the parts of this program is greater than the individual things"

Value of individual PC-PEP components

"I think the prostate cancer surgery… to me, the most devastating part of it was the depression and loss of sexual function. […] Well the two go hand and hand, if you don't have the ability to have an erection being depressed is really a double whammy because you're like ugh I can't do that and even if I could I probably wouldn't want to anyway because I'm just 'ugh blah' but anyway. […] I lost one part of intimacy, the program helped me bring back some of the other stuff I was doing in the past to replace it, kind of as a replacement."

"My wife and I have good communication, already and going through this experience has made it I think even better. And we joked a bit about the intimacy ideas and how to be more intimate with each other. The program has been extremely helpful in terms of opening up the communication.[…] I remember Dr. Gabriela spoke about the various

types of intimacy in which we can engage and my wife and I talk about it and I would say to her, in a joke, "I need the touch. I need the hug". And I certainly have been more expressive in my own demonstration. I've always been a tactile person anyway, but I think even more so since this program."

"It improved my relationship with my relatives that are long distance that I had to call, because I felt more open to talking. I thought yeah I really do have to do this because they knew I had prostate cancer but we never discussed it. It was kind of 'wish you well' kind of thing, 'how you doing? I'm doing fine. Of course, I'm doing fine, I'm a man, I'm always doing fine', It helped that out. It really helped me connect with them a bit better"

"One of the effects that I have been experiencing is overnight having erectile dysfunction where that has not been an issue for me before. And as a result of the hormonal medication, I feel like I've gone from 57 to 75 overnight. And my testosterone levels have dropped from 100% for someone at my age to 3%, overnight. So, emotionally I feel I've been much more fragile over the past couple of weeks. Throughout the course of this program [..] I realized I had more satisfaction at the start of the program than I do now, that the program is completed, because of the things I am going through, because of the way my body is readjusting. But I am thankful I was in the program to help alleviate some of the things that I am experiencing."

"… I think it made me realize that there are all kinds of ways you can connect with people","… I think with the intimacy, when you're able to put a handle on what you do on a day-to-day basis, you say "oh shit I was intimate with… intellectually. That was intimacy. That was connection"

"… You don't realize [there are] so many ways of being intimate. And. You're doing one and you're like "oh my god, that's one of the ones they taught me!"

"[I had] depression after my surgery and this program brought me out of it, and is keeping me out of it, because I have people to talk to now".

Buddy system

"The buddy system was fantastic, I'm down in the valley, there's no support group down there. The buddy system gave me someone to talk to that had the same condition I had and gave it a friendly accountability."

"I already knew about meditation, I knew about exercise, but I wasn't doing it. To be able to call somebody and say 'How're you doing Joe? […] How's your exercising going? Oh yeah, I did it four times out of five this week, and to be able to talk to them about the problems, talk to them about the Kegels, talk about urinary incontinence, talk about, and they'd say this exercise is hard, yeah it's hard on me too, it's phenomenal having that buddy system, it just puts your mind at ease. It's a friend, it's like a cancer buddy, prostate cancer buddy, 'PCB', you can talk about stuff, and you're calling them, you're keeping each other accountable, you're helping each other. Its, well it's great. I never would have had that, if it weren't for this program. I knew two people who had prostate cancer, or already had prostate cancer, in my church and I just couldn't find a way to approach them. You know… I don't know. I'm a man. I always consider myself fairly open and fairly communicative compared to maybe some other men but still it's not a topic, it's not a dinner topic conversation, sitting in church having tea, and I go 'How's your prostate cancer going?' [pretends to spit tea on ground]. PC-PEP created a safe environment for me to get the support and the camaraderie I needed and wanted. And the buddy system created the environment I needed. You can read all the books you want, all the University studies you want, you can read all that stuff, but it's not worth 10 minutes of sitting down with somebody that's already been through it and can look you in the eyes and say I've been through here's what you're going to experience, here's what you're going to feel. It makes all the difference in the world, that human connection."

"My buddy called me and he would say "How are you feeling?" And I would answer honestly, and I got a phone call from him just a few minutes later. So that support as men, has been really great. And we talk about intimate things, we talk about shrinkage, we talk about lack of sexual function, we talk about incontinence, and this has been great to have that with some other people. This can be very difficult for men, especially for men who never had that issue before. And I think of some of the men I met who had surgery, and have not had the nerve spearing approach, and right away suddenly, they are no longer able to have an erection, that can be a very devastating thing for people. And it kinda brings a dynamic of loss. And I would say that part of what my wife and I have gone through, for the past several months now, is grieving this loss. It's a funny thing, because I present well, I look healthy, but there is a loss still that we are going through. []The program helped me with this loss, by helping me feel healthier overall, and then having the wonderful interactions, the encouragement to be quiet, to meditate, to destress, to accept that somethings are out of our control, to rest in that has been a good thing for me."

Exercise

"This program has done a lot because I really felt the exercises that Rob demonstrated and the cardio, it just made you feel better, it gave you a purpose of doing it all"

"I lost at least 13-14 pounds during this program. I've been really motivated in terms of working out. One of my biggest concerns was that while on hormonal medication there is a bigger chance that I could gain a lot of weight and experience some of the symptoms women experience in menopause: carrying extra weight around the waist, hot flashes. This program has motivated me in terms of my physical fitness [..] through the exercises, and the encouragement to keep at these exercises,"

"According to my exercise tracker my resting heartbeat is 7 beats per minute less than it was before the start of the program. And, you know, I'm continuing on."

"It brought back my physicality, it got me off the couch … the emails or the texts to exercise got me back on my horse as far as exercising goes."

"I was relatively physically fit. I think with now doing the exercises, I found a few muscles that I wasn't really quite sure of. I think if I continue I will be a young Arnold Schwarzenegger soon".

"It's been so tremendously valuable to me and I really hope that there are ways found to enable more men and their loved ones to benefit from this type of support program that will enable them to lead better lives. Because, you know, the good thing about prostate cancer is that [it has a] 95% 10-year survival rate. But there's being alive and then there's living…and I think there's 95% of people that are alive – but I'm not sure what percentage of them are still really living. And if we can improve that percentage, I think that would be incredibly valuable".

Pelvic floor exercises (Kegels)

"The program has been a godsend, I had been taught Kegels, but I was only doing them 5-7 minutes a day, through this program there's a different series of Kegels and different exercises and they're lasting me about 15 minutes and doing them 3 times a day and I would say during this past month I'm about 90% dryer. So for some days I look at my pad and says "did I do anything in it or not? Maybe I could use it the next day?" so it's really good. Before coming here this morning I was out shoveling the driveway, dry, well we're well into the afternoon and I haven't leaked so far today. So thanks be to god for this program, things are really coming along, because that's one thing I prayed for every night, "Please Lord, let me be dry" it's been a nuisance".

"The thing was – when I was diagnosed I got a sheet of paper that said 'kegels'. And I tried, and I think I said before, I overdid it and I really had a problem with my rectum going into spasm, because I had done the wrong muscles. Whereas I didn't have any issue following your program."

"You know the variety of Kegels was good. Each week it was a little different. I think it was the third week where they were all the short ones and that was great, it was only 7 minutes long. The first were 14 minutes but the 14 minutes the second time went by quite quickly also, so I thought the variety in the Kegels was very very helpful and motivating."

"Now I was blessed that my incontinence wasn't really a problem, I was continent about a month after my surgery so the Kegels, I still did them but I didn't really need to do them."

Diet

"Something more. I think you should have almost have like a pamphlet or something to take home, anti-cancer foods or something like that … something more concrete."

Stress reduction meditation and breathing

"You know, that diet, exercise… all that I would have anticipated to be valuable. The meditation, I never would have, I wouldn't have told you up front, I wouldn't have said yeah that's going to be very useful."

"I didn't really appreciate how valuable the meditation practice would have been in helping me become more at peace with where I'm going and what my life is and content with where it's happening. I was always a glass half-full type of person. But this has really allowed me to recapture that."

"I found the program made me think about a whole lot of things, that I wouldn't of taken the time to think about. Things certainly in the areas of meditation, and I'm a bit more intrigued by the meditation component. Other ways of doing it, and how you get the best value out of it; keep the mind from wondering I suppose."

"One thing that really irritated me though was trying to mentally concentrate on things, and I was all over the place. Meditation to me was irritating but I've never done it before. Now if I reflect on it, I would say it probably is beneficial and I need to put more effort into it."

"I really liked the meditation. I jumped into that, it's the one aspect I really enjoyed and probably was most compliant with … it was a way of starting each day and was a way of dealing with some the other stressors that happened".

"I found that a little bit weak for me, because I meditate about an hour every day, but my meditation is focused on scripture"

"I did the meditation just as a sense of duty. I got nothing out of it at all."

Daily videos

"I knew what I was supposed to be doing before I even took this program, but to have someone remind me, the texting and stuff like that, that helped me a lot".

"I liked that the videos were short enough, they didn't take that long …Just enough motivation … you'd like to have something that's going to motivate you and get you moving along and that was, I think it was successful in doing that".

"I didn't find the daily reminders motivational at all because I had the motivation already others may have found them helpful."

"I would have liked to have seen, and this is my own opinion, a few less emails, like kind of coming at you all the time."

Accountability

"The exercises, and the encouragement to keep at these exercises, having some accountability with my buddies who give me a call and say "what have you been up to?"

"It's like a friend, it's like a cancer buddy…you're keeping each other accountable and you're helping each other".

Time of access to the program with regards to diagnosis and treatment

"I think that if you're diagnosed with prostate cancer, I think this program should be the first thing that I found out about. I wish it had been. I was fortunate enough that I'm post-surgery by a couple of months, so I'm happy with that."

"Yeah, agreed, more [resources] in [the] medical system and diet right from day 1 of diagnosis. Lots of obvious things."

"The program itself has been fantastic. Honestly, for me, it was a bit too late, some of it would have been beneficial if I had received it before the treatment and it might have even influenced what type of treatment I would have gone for."

"I was told that doing the Kegel exercise prior to the surgery wasn't going to do me any good, so I didn't, but now I discovered that maybe they would of done a lot of good."

"…talk to men and meet men that are in the same situation as me, I was diagnosed several years ago, I was on active surveillance, I wish I could have had this program then to prepare me for my prostate cancer surgery."

Duration of the program (28 days)

"And to me I think you need this as a lifestyle. This is more than a 28-days thing. Like this is your new life. And if people understand this then they would come to an awakening that I need to exercise and eat better … because in 10 years from now I'll be better."

"Oh I think it could become [my] lifestyle. Ongoing."

"I wouldn't want to go longer than 28 days, but then I like follow up later"
"Maybe with some type of maintenance afterwards or something. But it doesn't need to be as intense as this. 28 days is one of the things where you can make a habit out of. And maybe have something with maintenance after the programme. Like a 6-month check-up or something."

"... but if there were a periodic check-in like 6 months or something like that, then at the back of your mind you got, oh well okay I'm going to be held accountable – I'm going to be held accountable, and yeah I should keep on doing this, but without that then I might just slip back to..."

Perceived challenges to adhering to the PC-PEP program during the prescribed time.

Intensity of the program

"This is a boot camp. It's pretty intense what we just did.[…] It is a boot camp."

"It's pretty strenuous to keep up more than a month…"

Employment, lifestyle, and environmental factors

"I found compliance difficult sometimes, especially on the Kegels, your phone would go off, […] and I'd be in the elevator and start doing it and everyone would be looking at you."

"That's a comment to have for people that are working. I'm working still so fortunately I have flexibility in my work that's able to make it so I can go to the gym for this hour do my thing. […] For people who are working, this is a challenge".

"And this should be said to all men who are still working, I said this to my wife, thank God I'm retired because the time commitment for this. With all the exercise it took about an hour to do … so, I mean when you're working how would you find the time to do this".

"It's tough for me because I've got a really busy schedule, I work, I travel…But I did it anyway because I knew this is what I needed and it was good for me."

"And that's one of the problems, you have to do aerobic exercise but it's too cold to do that kind of thing in the month of January. And I know [participant] was running out there in –10 but that's not me".

Personal circumstances

"It was very poor for me, the physical intimacy. Well, my wife is 78 and she says, well, she's not interested in [physical intimacy] anymore".

"Well, if you have relationship problems it puts more emphasis on it… a reminder of the difficulties… if you're trying to forget about it and just get on, well that brings you right back, it's not going to go away. So now we had to look at what was not working and see what we could do about it. In a way it was good because it helped us both realize some things..."

"So I think for us 80-year-olds, the old connections are few and far between".

"I had looked up a couple of buddies and unfortunately found out they died. Especially, I had a very good friend in Montreal, saw him a year before always communication, I said I better check obituaries first, and sure enough".

"So whole lot of things I'd say good experience meeting a group of guys that are going through a similar situation, that's helpful. As guys we don't talk about a lot of stuff and it's hard to even talk to your friends it seems, especially if they haven't been through it. Overall it was a good experience".

"But like you said men don't talk".

"I found that I had trouble reaching out and sometimes I wouldn't do it. […] I found it awkward getting the timing right."
